# Composite cardiovascular risk and BMI affected comparative profiles of BIAsp 30 + metformin vs BIAsp 30 monotherapy: a MERIT post-hoc analysis

**DOI:** 10.1038/s41598-021-83410-x

**Published:** 2021-02-18

**Authors:** Lixin Guo, Baocheng Chang, Li Chen, Liyong Yang, Yu Liu, Bo Feng, Qinghua He

**Affiliations:** 1 Department of Endocrinology, Beijing Hospital, National Center of Gerontology; Institute of Geriatric Medicine, Chinese Academy of Medical Sciences, No. 1, Dahua Road, Dongcheng District, Beijing, 100730 China; 2 Tianjin Medical University Metabolic Diseases Hospital, Tianjin, China; 3 Department of Endocrinology, Qilu Hospital of Shandong University, Jinan, China; 4 The First Affiliated Hospital of Fujian Medical University, Fuzhou, China; 5 Sir Run Run Shaw Hospital of Nanjing Medical University, Nanjing, China; 6 Shanghai East Hospital Affiliated To Tongji University, Shanghai, China

**Keywords:** Diseases, Endocrinology, Medical research

## Abstract

We assessed whether comparative efficacy and safety of biphasic insulin aspart 30 (BIAsp 30) plus metformin versus BIAsp 30 monotherapy differed for patients with type 2 diabetes mellitus (T2DM) inadequately controlled with oral antidiabetic drugs with different cardiovascular risk scores and different body mass indexes (BMI) by performing a post hoc analysis of the randomized controlled MERIT study. In the MERIT study, eligible patients were randomized 1:1 to receive BIAsp 30 plus metformin or BIAsp 30 for 16 weeks. Patients in the 2 treatment groups were classified into “low” and “high” risk subgroups based on their GloboRisk scores and into “BMI ≤ 26 kg/m^2^”and “BMI > 26 kg/m^2^” subgroups. Primary efficacy endpoint was between-treatments comparison of HbA1c changes from baseline for these 2 sets of subgroups. Between-treatments comparisons of secondary efficacy and safety endpoints were also performed. We found that BIAsp 30 plus metformin led to significantly higher percentage of high-risk patients achieving HbA1c target < 7% than BIAsp 30 monotherapy, with an overall comparable safety profile for high-risk patients. Meanwhile, for patients with BMI ≤ 26 kg/m^2^, compared with BIAsp 30 monotherapy, BIAsp 30 plus metformin led to significantly higher percentages of patients achieving HbA1c target (47.83% vs 28.17%, *P* = 0.0165) and composite target of HbA1c < 7% without hypoglycemia or weight gain (20.29% vs 6.85%, *P* = 0.0187) and have a slightly better safety profile. In conclusion, for T2DM patients at high CV risk or with BMI ≤ 26 kg/m^2^, BIAsp 30 plus metformin was preferable to BIAsp 30 monotherapy.

## Introduction

Type 2 diabetes mellitus (T2DM) is a progressive disease characterized by continuing decline of pancreatic islet β-cell function and subsequent decrease in endogenous insulin secretion^1–3^. In China, T2DM had an estimated prevalence of 10.9% among adult in 2013^4–6^. Strict glycemic control by lowering glycated hemoglobin (HbA1c) is at the center of T2DM management^7,8^. The current American Diabetes Association (ADA) consensus statement recommends metformin as the preferred initial pharmacological treatment for T2DM^9^. It also recommends that metformin should be continued so long as it can be tolerated and not contraindicated and that other pharmacological agents such as insulin should be added to metformin^9^. In China, oral antidiabetic (OAD) monotherapy was the most common therapy (51.2%), followed by insulin in combination with OAD (27.0%) and insulin monotherapy (21.8%)^5,10^. In addition, most patients on insulin therapy in China received premixed insulin such as biphasic insulin aspart 30 (BIAsp 30)^10^. The 16-week randomized, parallel-controlled MERIT study was designed to mimic the real-life practice in China and to assess efficacy and safety of BIAsp 30 with and without metformin for patients with T2DM inadequately controlled with OADs^5^. It found that BIAsp30 plus metformin was not inferior to BIAsp 30 monotherapy in safely reducing HbA1c level, that BIAsp 30 plus metformin was associated with a significantly higher proportion of patients achieving HbA1c < 7% and achieving HbA1c < 7% without hypoglycemia or weight gain^5^.

ADA guidelines recommend a patient-centered approach in choosing proper T2DM treatment for individual patient, and one important consideration is cardiovascular (CV) comorbidities^9,11^. Cardiovascular disease (CVD) is the leading cause of death in T2DM patients, and the constantly evolving T2DM treatment algorithm includes better control of CV risk in T2DM patients at high CV risks^12^. It has been reported that metformin may reduce the risk of CV events and death^9^, as well as improve CV risk profile^7,13,14^. Meanwhile, numerous studies have assessed the effects of various CV risk factors such as total cholesterol (TC), body mass index (BMI) and blood pressure on efficacy of OADs such as metformin^15–20^. However, the effect of a patient’s composite CV risk score on comparative efficacy and safety of insulin with and without metformin has not been studied. GloboRisk is a tool for predicting a person’s10-year risk of CVD that can be recalibrated and updated for application in 182 countries with readily available information^21,22^. Unlike other risk scores such as the Framingham equation, GloboRisk allows the age patterns of CVD risk to vary by gender and across different populations and also allows for age-related attenuation of the effect of risk factors on CVD outcomes^21,22^. Laboratory-based GloboRisk includes smoking, systolic blood pressure (SBP), diabetes, TC, gender and age as variables^21,22^. A subject in a low and middle-income country such as China with ≥ 20% 10-year CVD risk is considered to have high CV risk^22^. One aim of the current study was to perform a post hoc analysis of the MERIT study to assess whether and how comparative efficacy and safety of BIAsp 30 with and without metformin differed for patients with different 10-year CVD risk score calculated based on laboratory-based GloboRisk.

BMI was not one of the variables included in the laboratory-based GloboRisk^21,22^. However, BMI is commonly considered an important CV risk factor^23^. Whether a patient’s baseline BMI affected efficacy of various OADs including metformin remains controversial^17–20^. One post hoc analysis of the MERIT study revealed similar HbA1c changes from baseline associated with BIAsp30 plus metformin treatment among the 3 BMI subgroups: normal weight (BMI, 18.5–23.9 kg/m^2^), overweight (BMI, 24.0–27.9 kg/m^2^) and obese (BMI, ≥ 28 kg/m^2^)^5^. In the current study, we did a preliminary analysis regarding whether comparative efficacy and safety of BIAsp 30 with and without metformin differed for patients with BMI ≤ 26 kg/m^2^ versus BMI > 26 kg/m^2^.

## Methods

### Design, participants and interventions

This was a post hoc analysis of the MERIT study, a 16-week, randomized, open-label, multicenter, parallel-controlled study conducted at 6 medical centers in China^5^. Details of the design and method of the MERIT study were described previously^5^. The MERIT study (registered at the Chinese Clinical Trial Registry (http://www.who.int/ictrp/network/chictr2/en/; CTR-IPR-15006834) assessed efficacy and safety of BIAsp 30 with and without metformin for patients with T2DM inadequately controlled with OADs. Briefly, patients 18–79 years old diagnosed with T2DM with BMI ≥ 18.5 kg/m^2^ and HbA1c ≥ 7% despite treatment with two or more OADs for more than 3 months from 6 medical centers in China were recruited for this study^5^. Details of the exclusion criteria were described previously^5^. The study was approved by the institutional review board of each participating center (Beijing Hospital, Tianjin Medical University Metabolic Diseases Hospital, Qilu Hospital of Shandong University, The First Affiliated Hospital of Fujian Medical University, Sir Run Run Shaw Hospital of Nanjing Medical University, and Shanghai East Hospital affiliated to Tongji University), and was conducted in accordance with the Declaration of Helsinki^24^ and Good Clinical Practice of China^25^. All patients provided written informed consent before any trial-related activities. The study was registered at the Chinese Clinical Trial Registry (http://www.who.int/ictrp/network/chictr2/en/): CTR-IPR-15006834.

The MERIT study consisted of a 2-week screening period and a 16-week treatment period, wherein the treatment period was divided into a 4-week titration period and a 12-week maintenance period. After screening, eligible patients were randomized 1:1 to receive BIAsp 30 (NovoMix30) subcutaneous injection plus oral metformin hydrochloride (Glucophage) or only BIAsp 30 subcutaneous injection. Both drugs were manufactured by Merck Serono Pharmaceutical R&D Co., Ltd (Beijing, China). Details of the BIAsp 30 and metformin adjustment and titration schedules and final doses were described previously^5^. Briefly, during the titration period, doses of metformin and insulin for each patient were adjusted to achieve fasting plasma glucose (FPG) target of 4.4–7.0 mmol/L and 2-h postprandial glucose (PPG) target of < 10 mmol/L (180 mg/dL). The initial dose of metformin during the titration phase was 500 or 850 mg once daily and the dose was titrated to a maximum tolerated dose of ≤ 2500 mg/day (850 mg administered 3 times daily). The initial doses of BIAsp 30 were 0.2 U/kg/day and 0.3 U/kg/day for the BIAsp 30 plus metformin group and the BIAsp 30 monotherapy group, respectively. The BIAsp 30 dose for the BIAsp 30 plus metformin group was titrated until optimal metformin dose was reached. For both treatment groups, the daily BIAsp 30 dose was split into 2 portions that were administered 5 min before breakfast and dinner. The dose of the pre-breakfast BIAsp 30 were adjusted according to FPG and post-breakfast PPG, and the dose of pre-dinner BIAsp 30 were adjusted according to and FPG and PPG before meals^5^.

### Patient populations, efficacy and safety endpoints of the present post hoc analysis

Laboratory-based GloboRisk score was used to calculate the 10-year CVD risk of each patient. China equation (http://www.globorisk.org/calc/labform) was used for GloboRisk score calculation. Variables included age, gender, TC, SBP, current smoking and diabetes^21,22^. The calculation could be performed at http://www.globorisk.org/calc/labform. As we did not record whether each patient in our study was a smoker or non-smoker, we ran the calculation twice, assuming all patients were smokers and then assuming all were non-smokers, and came up with 2 GloboRisk scores for each patient. Patients with ≥ 20% CVD risk were considered high-risk patients, while patients with < 20% CVD risk were considered low-risk patients^21,22^. Patients in the 2 treatment groups were classified into the low-risk and high-risk subgroups based on their GloboRisk scores. Additionally, patients in the 2 treatment groups were classified into the “BMI ≤ 26 kg/m^2^” and “BMI > 26 kg/m^2^” subgroups.

Primary efficacy endpoint was HbA1c changes from baseline compared between the 2 treatment groups (between-groups comparison) for the low-risk and high-risk subgroups as well as the “BMI ≤ 26 kg/m^2^” and “BMI > 26 kg/m^2^” subgroups.

Secondary efficacy endpoints included between-groups comparisons of percentages of patients who achieved HbA1c target (< 7%) and percentages of patients who achieved a pre-specified composite target (HbA1c < 7% without hypoglycaemia or weight gain) for the 2 pairs of subgroups.

Safety outcomes included between-groups comparisons of percentages of patients who had adverse events (AEs) and treatment-related adverse reactions, as well as between-groups comparisons of weight gains from baseline and percentages of patients who experienced at least 1 hypoglycemic episode and mean number of hypoglycemic episode(s) per patient for the 2 pairs of subgroups.

### Statistical analysis

Efficacy analyses were conducted on the intent-to-treatment (ITT) population (all randomized patients receiving ≥ 1 dose of study drug) with last observation carried forward (LOCF), while safety analysis were conducted on the safety set (SS) (all randomized patients who received ≥ 1 dose of study drug and had baseline safety data and safety data for at least 1 follow-up visit).

Data were presented as mean ± standard deviation (SD) or N (percentage). To evaluate between-groups differences, t test or Wilcoxon rank sum test was used for continuous variables while Chi-square test was used for categorical variables. Statistical significance was accepted with a *P* value < 0.05. SAS version 9.1.3 for Windows (SAS Institute Inc., Cary, North Carolina, USA) was used for all statistical analyses.

## Results

### For patients at high 10-year CV risk, compared with BIAsp 30 monotherapy, BIAsp 30 plus metformin led to significantly higher percentage of patients achieving HbA1c target of < 7%

**Figure 1:**
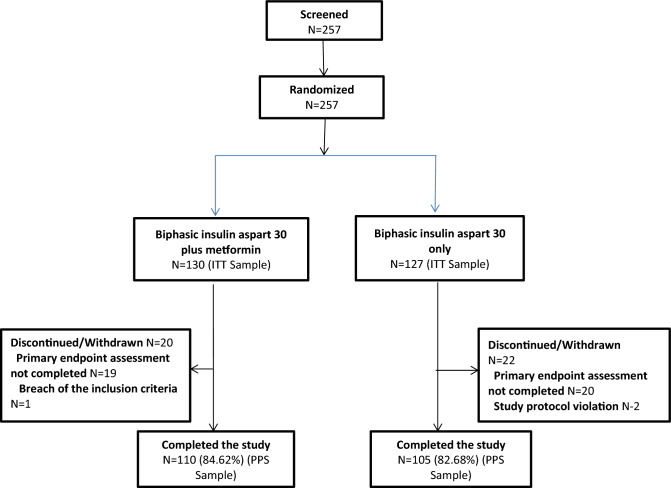
Flow diagram of the MERIT study. ITT, intent-to treat (created using Microsoft Word 2010, https://www.microsoft.com/en-us/microsoft-365/previous-versions/microsoft-word-2010).

Flow diagram of the MERIT study was depicted in Fig. 1. The ITT populations included 130 and 127 patients in the BIAsp 30 plus metformin group and the BIAsp 30 monotherapy group, respectively. Among them, records of 103 patients in the BIAsp plus metformin group and 107 patients in the BIAsp 30 monotherapy group were complete for the purpose of GloboRisk calculation. Assuming all patients were non-smokers, the BIAsp 30 plus metformin group included 39 patients at low 10-year CV risk and 64 patients at high 10-year CV risk, while the BIAsp 30 monotherapy group included 56 low-risk patients and 51 high-risk patients. Assuming all patients were smokers, the BIAsp 30 plus metformin group included 14 low-risk patients and 89 high-risk patients, while the BIAsp monotherapy group included 17 low-risk patients and 90 high-risk patients (Table 1).

**Table 1:** Efficacy outcomes in patients with low (< 20%) and high (≥ 20%) 10-year risk of cardiovascular disease receiving biphasic insulin aspart 30 (BIAsp 30) and metformin combination therapy or BIAsp 30 monotherapy (ITT).

Efficacy outcomes	Assuming all patient were non-smokers						Assuming all patients were smokers					
	Low risk			High risk			Low risk			High risk		
	BIAsp 30 + Met (N = 39)	BIAsp 30 (N = 56)	*p* value	BIAsp 30 + Met (N = 64)	BIAsp 30 (N = 51)	*p* value	BIAsp 30 + Met (N = 14)	BIAsp 30 (N = 17)	*p* value	BIAsp 30 + Met (N = 89)	BIAsp 30 (N = 90)	*p* value
HbA1c change from baseline, % (mean ± SD)	− 1.99 ± 1.62	− 1.26 ± 2.39	0.2820	− 1.69 ± 1.64	− 1.36 ± 1.88	0.3939	− 2.00 ± 1.41	− 0.51 ± 3.72	0.3534	− 1.77 ± 1.67	− 1.46 ± 1.69	0.3986
Patients who achieved HbA1c target (< 7%), n (%)	18 (56.25%)	18 (37.50%)	0.0986	30 (54.55%)	16 (34.78%)	0.0470*	8 (66.67%)	5 (33.33%)	0.1283	40 (53.33%)	29 (36.71%)	0.0381*
Patients achieving HbA1c < 7% without hypoglycemia or weight gain, n (%)	8 (25.00%)	8 (16.67%)	0.3613	12 (21.43%)	7 (14.58%)	0.3678	5 (41.67%)	2 (13.33%)	0.1850	15 (19.74%)	13 (16.05%)	0.5464

*ITT* intention-to treat, *LOCF* last observation carried-forward, *BIAsp 30* biphasic insulin aspart 30, *Met* metformin, *HbA1c* glycated hemoglobin, *SD* standard deviation.

*p < 0.05.

Regardless of whether we assumed all patients were non-smokers or smokers, the 2 treatments led to comparable HbA1c changes from baseline and comparable percentages of patients achieving the composite endpoint of HbA1c < 7% without hypoglycemia or weight gain in both low-risk patients and high-risk patients (All *P* > 0.05) (Table 1).

Meanwhile, regardless of whether we assumed all patients were non-smokers or smokers, BIAsp 30 plus metformin led to significantly higher percentage of patients achieving HbA1c target of < 7% than BIAsp 30 monotherapy in high-risk patients (54.55% vs 34.78%, *P* = 0.0470; and 53.33% vs 36.71%, *P* = 0.0381 assuming all were non-smokers and smokers, respectively), while comparable percentages of low-risk patients in the 2 treatment groups achieved HbA1c target of < 7% (*P* > 0.05) (Table 1).

### BIAsp 30 plus metformin and BIAsp 30 monotherapy had overall comparable safety profiles for both low-risk and high-risk patients

Safety analyses were performed on the SS which included 128 and 125 patients in the BIAsp 30 plus metformin and the BIAsp monotherapy groups, respectively. Among them, records of 101 and 106 patients in the BIAsp 30 plus metformin group and the BIAsp 30 monotherapy group were complete for the purpose of GloboRisk calculation, respectively.

Assuming all patients were non-smokers, the BIAsp 30 plus metformin group included 38 low-risk patients and 63 high-risk patients, while the BIAsp 30 monotherapy group included 55 low-risk patients and 51 high-risk patients. Assuming all patients were smokers, the BIAsp 30 plus metformin group included 14 low-risk patients and 87 high-risk patients, while the BIAsp monotherapy group included 16 low-risk patients and 90 high-risk patients (Table 2).

**Table 2:** Safety profiles of patients with low (< 20%) and high (≥ 20%) 10-year risk of cardiovascular disease receiving biphasic insulin aspart 30 (BIAsp 30) and metformin combination therapy or BIAsp 30 monotherapy (SS).

Safety parameters	Assuming all patient were non-smokers						Assuming all patients were smokers					
	Low risk			High risk			Low risk			High risk		
	BIAsp 30 + Met (N = 38)	BIAsp 30 (N = 55)	*p* value	BIAsp 30 + Met (N = 63)	BIAsp 30 (N = 51)	*p* value	BIAsp 30 + Met (N = 14)	BIAsp 30 (N = 16)	*p* value	BIAsp 30 + Met (N = 87)	BIAsp 30 (N = 90)	*p* value
Patients who had AEs n (%)	19 (50.00%)	28 (50.91%)	0.9313	33 (52.38%)	27 (52.94%)	0.9525	6 (42.86%)	9 (56.25%)	0.7152	46 (52.87%)	46 (51.11%)	0.8145
Patients who had treatment-related adverse reactions, n (%)	5 (13.16%)	6 (10.91%)	0.9972	15 (23.81%)	6 (11.76%)	0.0990	1 (7.14%)	4 (25.00%)	0.3359	19 (21.84%)	8 (8.89%)	0.0166*
Weight gain from baseline, kg (mean ± SD)	0.81 ± 3.31	0.86 ± 2.93	0.9333	0.25 ± 1.96	1.37 ± 1.95	0.0117*	0.31 ± 3.33	1.00 ± 3.57	0.6106	0.48 ± 2.42	1.13 ± 2.27	0.0758
Patients who experienced at least one hypoglycemic episodes, n (%)	8 (21.05%)	10 (18.18%)	0.7305	17 (26.98%)	15 (29.41%)	0.7742	2 (14.29%)	4 (25.00%)	0.6567	23 (26.44%)	21 (23.335)	0.6329
Number of hypoglycemia episode(s) per patient, n (mean ± SD)	0.24 ± 0.49	0.38 ± 1.30	0.8379	0.87 ± 2.61	0.65 ± 1.32	0.8009	0.14 ± 0.36	0.81 ± 2.26	0.4209	0.71 ± 2.25	0.46 ± 1.07	0.6475

*SS* safety set, *BIAsp 30* biphasic insulin aspart 30, *Met* metformin, *AEs* adverse events, *HbA1c* standard deviation, *GI* gastrointestinal, *AE* adverse event.

*p < 0.05.

Regardless of whether all patients were assumed non-smokers or smokers, the 2 treatment groups had comparable percentages of patients who had AE(s) and patients experiencing at least 1 hypoglycemic episode as well as comparable numbers of hypoglycemic episode per patients in both low-risk and high-risk patients (*P* all > 0.05) (Table 2).

Meanwhile, regardless of whether all patients were assumed non-smokers or smokers, low-risk patients in the 2 treatment groups had comparable percentages of patients experiencing treatment-related adverse reaction(s) as well as comparable weight gains from baseline (All *P* > 0.05) (Table 2).

High-risk patients in the BIAsp 30 plus metformin group had significantly less weight gain than patients in the BIAsp 30 monotherapy group assuming all patients were non-smokers (0.25 ± 1.96 kg vs 1.37 ± 1.95, *P* = 0.0117), whereas when we assumed all patients were smokers, high-risk patients in the 2 treatment groups had comparable weight gains (*P* > 0.05) (Table 2).

Finally, when we assumed all patients were smokers, significantly higher percentage of high-risk patients in the BIAsp plus metformin group experienced treatment-related adverse reactions than patients in the BIAsp monotherapy group (21.84% vs 8.89%, *P* = 0.0166), while when all patients were assumed non-smokers, the 2 treatment groups had comparable percentages of high-risk patients with treatment-related adverse reactions (*P* > 005) (Table 2).

### Compared with BIAsp 30 monotherapy, BIAsp 30 plus metformin led to significantly higher percentages of patients with BMI ≤ 26 kg/m^2^ achieving HbA1c target of < 7% and achieving the composite target of HbA1c < 7% without hypoglycemia or weight gain

The BIAsp 30 plus metformin group included 84 patients with BMI ≤ 26 kg/m^2^ and 46 patients with BMI > 26 kg/m^2^ in the ITT population, while the BIAsp 30 monotherapy group included 85 patients with BMI ≤ 26 kg/m^2^ and 42 patients with BMI > 26 kg/m^2^ in the ITT population (Table 3).

**Table 3:** The effect of body mass index (BMI) (≤ 26 kg/m^2^ or > 26 kg/m^2^) on efficacy outcomes in patients receiving biphasic insulin aspart 30 (BIAsp 30) and metformin combination therapy or BIAsp 30 monotherapy (ITT LOCF).

Endpoints	BMI ≤ 26 kg/m^2^			BMI > 26 kg/m^2^		
	BIAsp 30 + Met (N = 84)	BIAsp 30 (N = 85)	*p* value	BIAsp 30 + Met (N = 46)	BIAsp 30 (N = 42)	*p* value
HbA1c change from baseline, % (mean ± SD)	− 1.71 ± 1.72	− 1.21 ± 1.45	0.3273	− 1.78 ± 1.53	− 1.53 ± 2.89	0.8557
Patients who achieved HbA1c target (< 7%), n (%)	33 (47.83%)	20 (28.17%)	0.0165*	26 (61.90%)	18 (48.65%)	0.2366
Patients achieving HbA1c < 7% without hypoglycemia or weight gain, n (%)	14 (20.29%)	5 (6.85%)	0.0187*	12 (27.91%)	12 (32.43%)	0.6597

*BMI* body mass index, *ITT* intention-to-treat, *LOCF* last observation carried forward, *HbA1c* glycated hemoglobin, *SD* standard deviation.

*p < 0.05.

The 2 treatment groups had comparable HbA1c changes from baseline in both patients with BMI ≤ 26 kg/m^2^ and patients with BMI > 26 kg/m^2^ (both *P* > 0.05) (Table 3). On the other hand, for patients with BMI ≤ 26 kg/m^2^, compared with BIAsp 30 monotherapy, BIAsp 30 plus metformin led to significantly higher percentages of patients achieving HbA1c target of < 7% (47.83% vs 28.17%, *P* = 0.0165) and achieving the composite target of HbA1c < 7% without hypoglycemia or weight gain (20.29% vs 6.85%, *P* = 0.0187), though not for patients with BMI > 26 kg/m^2^ (both *P* > 0.05) (Table 3).

### A slightly better safety profile was associated with BIAsp 30 plus metformin than BIAsp 30 monotherapy for patients with BMI ≤ 26 kg/m^2^ and a slightly worse safety profile associated with BIAsp 30 plus metformin than BIAsp 30 monotherapy for patients with BMI > 26 kg/m^2^

The BIAsp 30 plus metformin group included 82 patients with BMI ≤ 26 kg/m^2^ and 46 patients with BMI > 26 kg/m^2^ in the SS, while the BIAsp 30 monotherapy group included 83 patients with BMI ≤ 26 kg/m^2^ and 42 patients with BMI > 26 kg/m^2^ in the SS (Table 4).

**Table 4:** Effect of body mass index (BMI) (≤ 26 kg/m^2^ and > 26 kg/m^2^) on safety profiles of patients receiving biphasic insulin aspart 30 (BIAsp 30) and metformin combination therapy or BIAsp 30 monotherapy (SS).

Endpoints	BMI ≤ 26 kg/m^2^ or > 26 kg/m^2^					
	≤ 26 kg/m^2^			> 26 kg/m^2^		
	BIAsp 30 + Met (N = 82)	BIAsp 30 (N = 83)	*p* value	BIAsp 30 + Met (N = 46)	BIAsp 30 (N = 42)	*p* value
Patients experiencing AEs, n (%)	40 (48.78%)	43 (51.81%)	0.6974	21 (45.65%)	22 (52.38%)	0.5282
Patients experiencing treatment-related adverse reactions	15 (18.29%)	10 (12.05%)	0.2633	9 (19.57%)	3 (7.14%)	0.0899
Weight gain from baseline, kg (mean ± SD)	0.75 ± 2.46	1.51 ± 2.40	0.0449*	− 0.35 ± 2.91	0.46 ± 2.41	0.3852
Patients who experienced at least one hypoglycemic episodes, n (%)	17 (20.73%)	24 (28.92%)	0.2239	12 (26.09%)	4 (9.52%)	0.0442*
Number of hypoglycemia episode(s) per patient, n (mean ± SD)	0.62 ± 2.28	0.69 ± 1.49	0.1866	0.46 ± 0.91	0.10 ± 0.30	0.0329*

*BMI* body mass index, *SS* safety set, *AEs* adverse reaction, *SD* standard deviation, *FPG* fasting plasma glucose.

*p < 0.05.

For patients with BMI ≤ 26 kg/m^2^, compared with BIAsp 30 monotherapy, the BIAsp 30 plus metformin treatment led to significantly smaller weight gain (0.75 ± 2.46 kg vs 1.51 ± 2.40, *P* = 0.0449), comparable percentages of patients experiencing AEs and treatment-related adverse reactions, comparable percentage of patients having hypoglycemic episode(s) and comparable mean number of hypoglycemic episode per patient (All *P* > 0.05) (Table 4).

Meanwhile, for patients with BMI > 26 kg/m^2^, compared with BIAsp 30 monotherapy, BIAsp 30 plus metformin led to significantly greater percentage of patients having hypoglycemic episode(s) (26.05% vs 9.52%, *P* = 0.0442) and significantly more hypoglycemic episode per person (0.46 ± 0.91 vs 0.10 ± 0.30, *P* = 0.0329), as well as comparable percentages of patients experiencing AEs and treatment-related adverse reactions and comparable weight gain from baseline (All *P* > 0.05) (Table 4).

## Discussion

In this post hoc analysis of the MERIT study, we found that for patients at high 10-year CV risk (GloboRisk score ≥ 20%), compared with BIAsp 30 monotherapy, BIAsp 30 with metformin had a somewhat better efficacy profile (significantly higher percentage of patients achieving HbA1c target of < 7%, comparable HbA1C change from baseline and comparable percentage of patients achieving the composite target of HbA1c < 7% without hypoglycemia or weight gain) and an overall comparable safety profile, while BIAsp 30 with and without metformin had comparable efficacy and safety profiles for patients at low 10-year CV risk. Meanwhile, compared with BIAsp 30 monotherapy, for patients with BMI ≤ 26 kg/m^2^, BIAsp 30 plus metformin led to significantly higher percentages of patients achieving HbA1c target of < 7% and achieving the composite target of HbA1c < 7% without hypoglycemia or weight gain, and comparable HbA1c change from baseline. It also had a better safety profile (significantly less weight gain) than BIAsp 30 monotherapy in patients with BMI ≤ 26 kg/m^2^. As for patients with BMI > 26 kg/m^2^, the two treatments had comparable efficacy profiles and BIAsp 30 monotherapy had a somewhat better safety profile than BIAsp with metformin (lower percentage of patients having hypoglycemic episode[s] and less hypoglycemic episode per patient associated with BIAsp 30 monotherapy).

Metformin is the preferred initial pharmacological treatment for T2DM^5,9^. It has been recognized that adding metformin to insulin allows for insulin dose reduction and could reduce insulin-associated side effects such as weight gain^9,26^, a point also made evident by the MERIT study^5^. In the MERIT study, during the treatment period, patients in the BIAsp 30 plus metformin group received significantly lower daily insulin dose than patients in the BIAsp 30 monotherapy group (0.38 ± 0.14 U/kg/day vs 0.47 ± 0.15 U/kg/day) as well as experienced significantly less weight gain (0.33 ± 2.68 kg vs 1.16 ± 2.45 kg), although the 2 group of patients experienced comparable number of hypoglycemic events^5^. The MERIT study and others have shown that insulin plus metformin was both effective and safe^5,26–29^. Removing metformin from insulin—metformin combination therapy led to significant deterioration of glycemic control^26,30^.

An individualized, patient-centered and multi-factorial approach in managing T2DM is recommended, and better control of CV risk is one important feature of this approach^12,14^. Besides glycemic control, it was also important to modify other CV risk factors such as obesity, high blood pressure and hyperlipidemia in order to reduce risk of macrovascular and microvascular complications commonly associate with T2DM^14^. Therefore, the effects of various antidiabetic agents on CV risk factors should be considered when choosing a treatment for T2DM patients^9,14^. Studies have shown that metformin could improve CV risk profile in patients with T2DM as reflected by its ability to reduce TC, triglyceride, BMI, waist circumference and blood pressure^7,13,14,26^. Knowing that metformin could confer some cardiovascular benefits, there is still the question of exactly what kind of patients could benefit most from metformin. It has been reported that high blood pressure and hypertension phenotype suppressed the efficacy of metformin in lowering HbA1c^15^. A lower baseline TC level was reported to be a predictor for higher HbA1c level 6 months after metformin initiation, while at 18 months, baseline cholesterol level ceased to be a predictor^16^. At the same time, studies assessing whether a patient’s baseline BMI affected efficacy of metformin generated inconsistent results^17–20^. However, whether a patient’s composite CV risk affected efficacy and safety of metformin or comparative efficacy and safety of insulin with and without metformin has not been assessed, such a study could provide a comprehensive picture as to what kind of patients would benefit most from a particular treatment and could potentially help clinicians choose a treatment tailored to individual patient’s CV risk.

Our post hoc analysis of the MERIT study was the first to use GloboRisk, a tool for predicting a person’s 10-year risk of CVD^21,22^, to assess whether a patient’s composite CV risk affected comparative efficacy and safety of 2 treatments. Our results indicated that for patients with high CV risk, BIAsp 30 plus metformin combination therapy was preferable to BIAsp 30 monotherapy. For patients with low CV risk, the two treatments were comparable. Prescribing BIAsp 30 plus metformin combination therapy instead of BIAsp 30 monotherapy to patients at high CV risk could also take advantage of metformin’s known ability to improve a patient’s CV risk profile^7,13,14,26^. Our analysis demonstrated that GloboRisk could be a useful tool to help clinicians choose a proper treatment for patients according to their composite CV risk.

Laboratory-based GloboRisk does not include a patient’s baseline BMI as a variable^21,22^, although BMI is a commonly known CV risk factor^23^. Therefore we also assessed whether comparative efficacy and safety of BIAsp 30 with and without metformin differed for patients with BMI ≤ 26 kg/m^2^ and patients with BMI > 26 kg/m^2^ and found that BIAsp 30 plus metformin had better efficacy as well as led to significantly less weight gain than BIAsp 30 monotherapy for patients with BMI ≤ 26 kg/m^2^, while the 2 treatment had comparable efficacy profiles for patients with BMI > 26 kg/m^2^. Although this result seemed to somewhat differ from our findings that BIAsp 30 plus metformin had better efficacy than BIAsp 30 monotherapy for patients at high CV risk, it must be noted that although high BMI is a CV risk factor, high BMI alone does not represent high composite CV risk. Of course, more studies are needed.

What are the clinical implications of our findings? Our results suggested when choosing between BIAsp 30 plus metformin and BIAsp 30 monotherapy, a clinician could take into consideration a patient’s CV risk such as laboratory-based Globorisk score and BMI. For patients at high CV risk and/or with BMI ≤ 26 kg/m^2^, BIAsp 30 plus metformin combination is preferred, while for patients with low CV risk and/or BMI > 26 kg/m^2^, either treatment could be prescribed. Our analysis and finding could be especially helpful for optimizing treatment protocols for Chinese patients. Due to social, economic and psychological factors, delay in insulin initiation and insulin overuse are both common in China^31–35^. The Observational Registry for BI Treatment (ORBIT) on 19,894 patients with T2DM in China found that prior to insulin initiation, mean duration of diabetes was 6.4 ± 5.3 years and mean HbA1c level was 9.6 ± 2.0%^32^. In addition, patients with shorter T2DM duration at insulin initiation might also have better glycemic control than patients with longer T2DM duration^10^. The MERIT study underlying our analysis included patients with a mean T2DM duration of approximately 9 years and HbA1c level of around 9% prior to insulin initiation, and as such it reflected the real-world practice in China^5^. As it was known that better glycemic control could reduce a patient’s CV risk^13,14^ and long T2DM duration itself was an additional CV risk factor for patients with diabetes^36^, patients deemed to be at high CV risk according to their GloboRisk scores (≥ 20%) would have even higher CV risk if they have poor glycemic control and/or long T2DM duration, for these patients, BIAsp 30 and metformin combination therapy could potentially be especially advantageous. Of course, our study is a preliminary analysis, more researches are needed to further elucidate the role of T2DM duration in comparative profiles of BIAsp 30 and metformin combination therapy and BIAsp 30 monotherapy.

Our study was limited by the fact that it was a post hoc analysis of a randomized controlled study not originally designed to test whether a patient’ composite CV risk score and BMI affected comparative efficacy and safety of BIAsp 30 with and without metformin, therefore, our results might not carry the same weight as results obtained from a perspective, pre-specified analysis, and should be viewed as being hypothesis-generating and needing further confirmation by perspective pre-specified studies. Secondly, since we did not record whether our patients were smokers or non-smokers, we assumed all patients were smokers and then all were non-smokers and calculated their GloboRisk scores twice, therefore the accuracy of our results regarding the effect of patient’s CV risk on comparative efficacy and safety of BIAsp with and without metformin could be reduced. However, since regardless of whether all patients were assumed smokers or non-smokers, we got consistent results regarding comparative efficacy of the 2 treatments, we think our findings were solid. Thirdly, as a post hoc analysis, the sample size of our study was not pre-calculated to make sure it was adequately powered. In addition, the MERIT study underlying our analysis was a medium-term 16-week study rather than a long-term study^5^, therefore our post-hoc analysis only address the impact of CV risk and BMI on comparative medium-term efficacy and safety profiles of the 2 treatments. 3 months are generally enough to properly assess the efficacy and safety of an antidiabetic treatment, and antidiabetic studies lasting 3–4 months were common^26,28,29,37^. Of course, a long-term study could better address the long-term efficacy and safety of an antidiabetic treatment, however, due to limited funding, we decided that a 16-week study was proper for our purpose of comparing the 2 treatment regimens. On the other hand, our study was the first to use GloboRisk to analyze whether and how patient’s composite CV risk affected comparative efficacy and safety of BIAsp 30 with and without metformin, such a study could help a clinician choose a tailored treatment for a patients from several available treatment regimens according to the patient’s CV risk and/or BMI.

In conclusion, for Chinese patients at high CV risk (≥ 20% GloboRisk score) and/or with BMI ≤ 26 kg/m^2^, BIAsp 30 and metformin combination therapy could be preferable to BIAsp 30 monotherapy as BIAsp 30 and metformin combination therapy had a better efficacy profile as well as a comparable or even better safety profile than BIAsp 30 monotherapy. Meanwhile, for Chinese patients at low CV risk and/or with BMI > 26 kg/m^2^, either treatment could be prescribed, as the 2 treatment regimens had comparable efficacy profiles, although BIAsp 30 monotherapy had a slightly better safety profile than the combination therapy for patients with BMI > 26 kg/m^2^.

## Data availability

The data used for this manuscript are available on reasonable request from the corresponding author.
